# Skin-permeable gold nanoparticles with modifications azelamide monoethanolamine ameliorate inflammatory skin diseases

**DOI:** 10.1186/s40364-024-00663-0

**Published:** 2024-10-09

**Authors:** He Zhao, Han Zhao, Yan Tang, Mengfan Li, Yisheng Cai, Xin Xiao, Fanping He, Hongwen Huang, Yiya Zhang, Ji Li

**Affiliations:** 1 Department of Dermatology, Xiangya Hospital, Central South University, Changsha, China; 2 Hunan Key Laboratory of Aging Biology, Xiangya Hospital, Central South University, Changsha, China; 3 National Clinical Research Center for Geriatric Disorders, Xiangya Hospital, Central South University, Changsha, Hunan 410008 China; 4 College of Materials Science and Engineering, Hunan University, Changsha, Hunan 410082 P. R. China

**Keywords:** Gold nanoparticles, Azelamide monoethanolamine, Skin inflammation, Topical therapeutics

## Abstract

**Background:**

Traditional topical drug delivery for treating inflammatory skin diseases suffers from poor skin penetration and long-term side effects. Metal nanoparticles show promising application in topical drug delivery for inflammatory skin diseases.

**Methods:**

Here, we synthesized a new type of nanoparticles, azelamide monoethanolamine-functionalized gold nanoparticles (Au-MEA NPs), based on citrate-capped gold nanoparticles (Au-CA NPs) via the ligand exchange method. The physical and chemical properties of Au-CA NPs and Au-MEA NPs were characterized. In vivo studies were performed using imiquimod-induced psoriasis and LL37-induced rosacea animal models, respectively. For in vitro studies, a model of cellular inflammation was established using HaCaT cells stimulated with TNF-α. In addition, proteomics, gelatin zymography, and other techniques were used to investigate the possible therapeutic mechanisms of the Au-MEA NPs.

**Results:**

We found that Au-MEA NPs exhibited better stability and permeation properties compared to conventional Au-CA NPs. Transcutaneously administered Au-MEA NPs exerted potent therapeutic efficacy against both rosacea-like and psoriasiform skin dermatitis in vivo without overt signs of toxicity. Mechanistically, Au-MEA NPs reduced the production of pro-inflammatory mediators in keratinocytes by promoting SOD activity and inhibiting the activity of MMP9.

**Conclusion:**

Au-MEA NPs have the potential to be a topical nanomedicine for the effective and safe treatment of inflammatory skin diseases.

**Supplementary Information:**

The online version contains supplementary material available at 10.1186/s40364-024-00663-0.

## Introduction

Psoriasis and rosacea are common chronic inflammatory skin diseases in everyday life with high prevalence worldwide and many negative effects on patients’ quality of life [1–3]. To date, treatment options for such diseases are extensive. Transdermal drug delivery is a simple, non-invasive method of self-administration and avoids the risk of systemic side effects, making it a favorable option for the treatment of inflammatory skin diseases [4–6]. Despite advances in transdermal drug delivery, traditional topical delivery methods continue to be challenged by poor skin penetration efficacy and long-term side effects [6, 7]. Therefore, there is a need to develop a valid and safer topical treatment regimen.

Oxidative stress plays an important role in the pathogenesis of many inflammatory skin diseases, including psoriasis and rosacea [8, 9]. Excessive reactive oxygen species (ROS) accumulation reduces the skin’s physiological antioxidant capacity and damages the redox system of the epidermal microenvironment [10]. As a key signaling molecule, ROS regulates the inflammatory signals of keratinocytes by promoting the production of pro-inflammatory factors and activating nuclear factor kappa B (NF-κB) pathways [11]. Studies have reported that Quercetin can effectively remove ROS and treat psoriasis by increasing the activity of antioxidant enzymes [12]. Superoxide dismutase (SOD) is a widely distributed antioxidant enzyme that plays an important role in the fight against ROS-mediated oxidative stress diseases [13, 14]. Our previous study showed that inhibition of SOD can lead to ROS accumulation and exacerbate inflammatory skin diseases [15]. Matrix metalloproteinases (MMPs) are enzymes with specific biological activity involved in regulating inflammatory responses and are often used as an inflammatory marker for diagnosing and treating some inflammatory diseases [16–18]. MMP9 was reported to be elevated in the skin lesion of both rosacea and psoriasis and acts as a key player in the pathogenesis and progression of these skin diseases [19]. Pharmacological inhibition of MMP9 reduces psoriasiform and rosacea-like dermatitis [20, 21]. These previous studies clearly indicate that targeting SOD and MMP9 enzymatic activity could be an effective alternative for treating rosacea and psoriasis.

Nanoparticles (NPs) have demonstrated advantages in immunomodulating, vaccinating and treating inflammatory disorders due to their controllable size, shape and other unique physicochemical characteristics [22–25]. Notably, NPs possessing superior antioxidant capacity are capable of scavenging ROS and thus modulating the local microenvironment and suppressing inflammation [26, 27]. Gold nanoparticles (AuNPs) were found to possess SOD enzymatic activity that effectively scavenge excess ROS and modulate inflammation to alleviate drug-induced liver injury [28]. However, its transdermal properties and stability severely limit its use in inflammatory skin diseases [29, 30]. In fact, in vivo skin penetration can be improved by surface modification of metal nanoparticles [31]. Therefore, by surface modification of AuNPs, we tried to improve their transdermal properties and stability. Azelamide monoethanolamine (MEA) is an anionic surfactant that is widely used as a surfactant in cosmetics, but the biological function of azelamide MEA for the enhancement of AuNPs has not been reported.

In this study, we combined azelamide MEA with gold nanoparticles by simple ligand exchange to develop Au-MEA NPs, the new biological nanomaterials with positive biological functions. We found that Au-MEA NPs have good stability and permeation compared with conventional Au-CA NPs. Transdermal application of gel containing Au-MEA NPs could treat psoriasis and rosacea in mice, and was more effective than Au-CA NPs gel. And we further found that Au-MEA NPs attenuated inflammatory response by promoting the activity of SOD and reducing the activity of MMP9. Thus Au-MEA NPs show great translational potential for clinical application in inflammatory skin diseases.

## Materials and methods

### Synthesis of materials

#### Synthesis of Au-CA NPs

As study reported, the classical method based on the reduction of an Au (III) precursor with sodium citrate in an aqueous solution near the boiling point [32]. During the reaction, citric acid reduces gold ions (Au³⁺) from chloroauric acid (HAuCl₄) to neutral gold atoms (Au⁰). Furthermore, the reduction of HAuCl_4_ by sodium citrate synthesized Au-CA NPs with a regular size distribution and spherical shape. Briefly, 25 mL of 0.5 mM HAuCl_4_ solution was heated at 105℃ in a 100 mL three-neck round-bottom flask for 20 min under vigorous stirring. 0.588 mL of 170 mM sodium citrate was injected rapidly. After a few minutes the colorless solution became wine red. With another 20 min passed, the reaction was finished. Centrifuge at 12,000 rpm for 5 min and disperse product in deionized water.

#### Synthesis of Au-MEA NPs

Au-MEA NPs were synthesized based on ligand exchange of Au-CA NPs, which aimed to change citrate to azelamide MEA. This ligand-exchange process was performed by a simple method. Based on principle of concentration gradients, 542.6 mM azelamide MEA solution at 30 ℃ was added to a previously synthesized Au-CA NPs dispersion (concentration around 2.05 mM), in a ratio of 1:1 v/v. Then, the mixture was incubated with uniform agitation for around 24 h at 30 ℃ to allow the incorporation of the azelamide MEA ligands on the surface of the AuNPs. And the wine red turned blue. Finally, the sample was centrifuged at 12,000 rpm for 5 min. Then, the supernatant was discarded, and the solid was dispersed into deionized water.

#### Synthesis of FITC- Au-CA NPs and FITC- Au-MEA NPs

As previous study reported, 0.35 mL of 0.01 M 3-mercaptopropionic acid (MPA) aqueous solution was added to 4 mL of Au-CA NPs or Au-MEA NPs solution at pH 11. Then, the solid of Au-CA@MPA and Au-MEA@MPA was dispersed in deionized water after centrifugation. N-(3-Dimethylaminopropyl)-Nʹ-ethylcarbodiimide hydrochloride (EDC) (0.81 mg) and N-hydroxysuccinimide (NHS) (1.2 mg) were added to Au-CA NPs or Au-MEA NPs solution. After 30 min, methoxypolyethylene glycol amine (mPEG-NH_2_) terminated (10.4 mg) was added and the mixture was stirred for 24 h. Finally, fluorescein isothiocyanate (FITC) (0.2 mg) which was dispersed in 200 µL dimethylformamide was added to 4 mL mixture (37 ℃ for 24 h). The solvent was removed under high speed centrifugation to achieve a final gold nanoparticles.

### Production of Au-CA NPs gel and Au-MEA NPs gel

For preparation of Au-CA NPs gel and Au-MEA NPs gel, SEPIMAX ZEN (10 mg/mL) mixed with the suspension of Au-CA NPs (0.404 mg/mL) and Au-MEA NPs (0.404 mg/mL) which were dispersed by ultrasonic waves. Homogeneous gels were formed under vigorous stirring at rate of 900 rpm. The blank gel was produced as same way without doping with suspension of Au-CA NPs or Au-MEA NPs.

### Materials characterization

Structural and size analyses of the gold nanoparticles were performed using transmission electron microscopy (TEM, JEOL JEM 2100Plus, Japan). Rigaku Miniflex-600 diffractometer (Japan) was used for obtaining an XRD pattern of the synthesized Au-CA NPs and Au-MEA NPs. UV-visible spectra were recorded on a Tu-1901 UV-Vis spectrometer (China) in a range of 400–800 nm wavelength. The Cu Kα source radiation with λ = 1.54 Å was operated at a voltage of 40 kV. The organic compounds conjugated on the surface of the AuNPs were classified using Fourier transform infrared spectroscopy (FTIR, Thermo Fisher Scientific Nicolet iS20, USA) spectroscopy in the range of 400–4000 cm^− 1^. XPS studies were performed on a Thermo Fisher Scientific K-Alpha spectrometer (Al-Ka radiation, 1486.6 eV). Spectra were recorded in the constant pass energy mode at 100 eV, using a 400 μm diameter analysis area. The size and dispersal nature of the nanoparticles and measurement of zeta potential were determined using a dynamic light scattering (DLS, Zetasizer Nano series, Malvern, UK) particle analyzer. The surface morphologies of Au-CA NPs gel, Au-MEA NPs gel and Blank gel were examined by a Sigma HD field emission SEM (Carl Zeiss, Germany), followed by EDS analysis to assess their elemental constitutions.

### Cell lines and culture

HaCaT (human keratinocytes) cell line was obtained from the American Type Culture Collection (ATCC; Manassas, VA, USA) and was cultured in Dulbecco’s modified Eagle medium (DMEM; Gibco, Grand Island, NY, USA) supplemented with 10% fetal bovine serum (FBS; Gibco) and 1% penicillin/streptomycin under a humidified atmosphere containing 5% CO_2_ at 37 °C. For tumor necrosis factor alpha (TNF-α; Thermo Fisher Scientific, 300–01 A, USA) treatment, HaCaT cells were starved overnight, and then incubated with Au-CA NPs or Au-MEA NPs for 12 h before TNF-α (100 ng/mL) stimulation. The mRNA levels or protein levels were measured 12 or 24 h after TNF-α stimulation, respectively. The levels of phosphorylated proteins and associated total proteins were detected immediately after 30 min of TNF-α stimulation. All experiments were performed at least three times.

### Animals

Female BALB/c mice (6 to 7 weeks old, weight 16–21 g) were purchased from SLAC Laboratory Animal Co., Ltd. (Shanghai, China). All mice were bred, housed, and used under specific pathogen-free conditions. All animal experiments were approved by the Animal Ethics Committee of the Xiangya Hospital of Central South University (Authorization number 201611610).

### ICP-MS analysis

HaCaT cells were collected after incubated Au-CA NPs or Au-MEA NPs (both 25 µg/mL) for around 24 h and stored in 4% paraformaldehyde (PFA). At 10 days after external application of Au-CA NPs gel and Au-MEA NPs gel, mice were euthanized, and skin and major organs were harvested. Cells and tissues were digested by mixture of HNO_3_ and HCl (1:3/v: v) for around 12 h and diluted 20 fold with deionized water. Total amount of gold was measured by ICP-MS (Agilent ICP-MS7900, USA).

### Cell viability assay

HaCaT cells at a density of 2 × 10^3^/well were seeded in a 96-well plate and incubated overnight. Then, the cells were pretreated with AuNPs at different concentrations (0–100 µg/mL) for 24 h. CCK-8 solution was added into the medium and incubated for 1 h. The absorbance was measured at 450 nm by a microplate reader (PerkinElmer EnSight).

### Mouse model induction and treatment

For the imiquimod-induced psoriasis model, a daily topical 5% IMQ cream (Sichuan MED-SHINE Pharmaceutical Co., Ltd) was applied to the right ear of mice from days 3 to 8. An equivalent amount of petroleum jelly (Vaseline; Unilever, London, UK) was topically applied to the left ear of mice for the disease-free control group. Mice were randomly divided into four groups. Azelamide MEA gel (MEA gel), Au-CA NPs gel, Au-MEA NPs gel (25 mg) or an equal volume of gel (Blank gel) was topically applied to each ear three days before IMQ cream was applied and topically applied for nine consecutive days.

For the rosacea-like skin lesion, mice received a daily topical dose of treatment or control gel on the shaved skin of their back for five consecutive days. LL37 peptide was injected intradermally for the last two days to induce rosacea-like skin lesions as previously described [33].

### Histological analysis

Mouse ear and dorsal skin samples, as well as the heart, liver, spleen, lungs and kidneys were previously fixed in 10% formalin. The fixed tissues were dehydrated in gradient alcohol and xylene. Then 6 μm skin sections were sliced using a microtome (Leica, Germany) and stained with H&E based on standard protocols.

### TEM of the HaCaT cells and skin tissues

HaCaT cells and skin tissues were fixed in 2.5% glutaraldehyde at 4℃ overnight and postfixed in 1% osmium tetroxide for 1 h. After washing with phosphate buffer, a series of graded acetones were used for dehydration. Samples were embedded in araldite resin. Ultrathin sections of ~ 70 nm were stained with 3% uranyl acetate and lead nitrate for observation with the Hitachi HT7700 TEM.

### Quantitative real-time polymerase chain reaction

Cells and tissues were lysed in Trizol (Invitrogen Life Technologies, USA). 1 µg RNA was transcribed to cDNA by using the Maxima H Minus First-Strand cDNA Synthesis Kit with dsDNase (Thermo Fisher Scientific, K1682, USA). Real-time PCR was performed using qPCR SYBR Green Master Mix (Vazyme Biotech Co., Ltd., Nanjing, China) and signal detection was performed in triplicate using CFX Connect Real-Time System (Bio-Rad). The sequences of the primers are listed in Table 1.

**Table 1:** List of primers used for real-time PCR

Target gene	Forward primers	Reverse primers
Human GAPDH	TGTTGCCATCAATGACCCCTT	CTCCACGACGTACTCAGCG
Human IL6	CCTGAACCTTCCAAAGATGGC	TTCACCAGGCAAGTCTCCTCA
Human IL1B	AGCTACGAATCTCCGACCAC	CGTTATCCCATGTGTCGAAGAA
Human TNF	CCCATGTTGTAGCAAACCCTC	TATCTCTCAGCTCCACGCCA
Human IL8	TTTTGCCAAGGAGTGCTAAAGA	AACCCTCTGCACCCAGTTTTC
Human CCL2	CAGCCAGATGCAATCAATGCC	TGGAATCCTGAACCCACTTCT
Human CCL20	TGCTGTACCAAGAGTTTGCTC	CGCACACAGACAACTTTTTCTTT
Human CXCL10	GTGGCATTCAAGGAGTACCTC	TGATGGCCTTCGATTCTGGATT
Human MMP9	AGACCTGGGCAGATTCCAAAC	CGGCAAGTCTTCCGAGTAGT
Mouse Gapdh	AGGTCGGTGTGAACGGATTTG	TGTAGACCATGTAGTTGAGGTCA
Mouse Il17a	TCCAGAATGTGAAGGTCAACC	TATCAGGGTCTTCATTGCGG
Mouse Il17f	GGAGGTAGCAGCTCGGAAGA	GGAGCGGTTCTGGAATTCAC
Mouse Il22	AGCTTGAGGTGTCCAACTTC	GGTAGCACTGATCCTTAGCACTG
Mouse Il23a	CCCGTATCCAGTGTGAAGATG	GGCTCCCCTTTGAAGATGTC
Mouse Il1b	GCAACTGTTCCTGAACTCAACT	ATCTTTTGGGGTCCGTCAACT
Mouse Il6	TAGTCCTTCCTACCCCAATTTCC	TTGGTCCTTAGCCACTCCTTC
Mouse Tnf	CTGAACTTCGGGGTGATCGG	GGCTTGTCACTCGAATTTTGAGA
Mouse Vegf	TATTCAGCGGACTCACCAGC	AACCAACCTCCTCAAACCGT
Mouse Cxcl2	CGCTGTCAATGCCTGAAGAC	ACACTCAAGCTCTGGATGTTCTTG

### Immunofluorescence

Fresh skin tissues from mice were embedded by optimal cutting temperature compound. 6 μm skin tissue sections or cells grown on round glass coverslips in 24-well plates were fixed with 4% PFA. 5% donkey serum was used to block sections for 1 h after permeabilized by 0.3% Triton X-100 at room temperature for 15 min. Then anti-CD4 (Thermo Fisher Scientific Cat# 14-0042-85, 1:100), anti-CD31 (BD Biosciences Cat# 558736, 1:100), anti-Ki67 (Thermo Fisher Scientific Cat# 14-5698-80, 1:500) and anti-p65 (Cell Signaling Technology Cat# 8242, 1:200) antibodies were administered overnight at 4 ℃. The unbound antibody from tissue sections were washed with PBS and sections were incubated with the corresponding fluorescent secondary antibodies (1:500 dilutions) at room temperature for 1 h, and then sections were washed and mounted with 4′, 6-diamidino-2-phenylindole (DAPI) for 3 min. Finally, the proteins expression was observed under Nikon DS-Ri2 (Nikon, Japan).

### Western blot assays

Proteins of HaCat cells were extracted with RIPA lysis buffer and were used for quantification with a BCA protein assay kit (Thermo Fisher Scientific, USA). The proteins were subjected to SDS-PAGE electrophoresis and transferred onto polyvinylidene fluoride (PVDF) membranes, which were blocked with 5% non-fat milk. Then PVDF membranes were immunoprobed with the corresponding primary antibodies at 4 °C overnight. Primary antibodies including anti-p65 (Cell Signaling Technology Cat# 8242, 1:1000), anti-phospho-p65 (Cell Signaling Technology Cat# 3033, 1:1000), anti-MMP9 (Abcam Cat# ab76003, 1:1000) and anti-GAPDH (Bioworld Technology Cat# AP0066, 1:15000) antibodies. The second day, PVDF membranes were incubated with secondary horseradish peroxidase-conjugated antibodies (1:10000 dilutions) at room temperature for 1 h.

### ROS measurements

The cells were collected and suspended in diluted DCFH-DA (1:1000, Beyotime Institute of Biotechnology). These cells were incubated in a cell incubator at 37 ℃ for 20 min and mix upside down every 3–5 min. Washing the cells with serum-free cell culture medium for three times. Fluorescence intensity was measured by a Zeiss Axio Scope A1 (Zeiss, Germany) following the manufacturer’s protocol.

### Gel Zymography

To test the enzymatic activity of MMP9, we performed zymography as previously described [34] with minor modifications. Briefly, conditioned media were separated on 10% polyacrylamide gels containing 1 mg/mL gelatin under nonreducing conditions. Gels were washed with eluent (TBS, 25% Triton X-100), followed by washing buffer (TBS, 5 mM CaCl_2_, 1 µM ZnCl_2_) and incubated overnight in incubation buffer (50 mM Tris–HCl pH 7.5, 10 mM CaCl_2_, 1 µM ZnCl_2_, 0.02% Brij-35) at 37 ℃. Gels were stained with Coomassie blue and visualized after decolorization.

### Hemolysis test

The hemolysis test was performed by mice blood sample from BALB/c mice. Erythrocytes were collected via centrifugation at 3000 rpm for 10 min. 400 µL of Au-CA NPs and Au-MEA NPs dispersed in saline at different concentration was added into 200 µL of the erythrocyte dilution (1:20 diluted with saline). Saline is negative control, while Triton is positive control. The solution was incubated for 2 h at 37 °C. Finally, the percentage of hemolysis was measured by microplate reader at 540 nm absorbance after centrifugation at 13,000 rpm for 10 min.

### LC-MS/MS analysis

Samples of mouse skin tissues protein (15 µg) were collected and processed according to the kit instructions (Omicsolution, China). Peptides were then separated using a Vanquish Neo HPLC system on a Thermo NanoVipe-C18 column (25 cm × 75 mm) and analyzed using an Orbitrap Exploris 480 mass spectrometer (Thermo Fisher Scientific, USA). DIA raw data were processed using Spectronaut (v18).

### Proteomics analysis

DIA raw data were processed using the “directDIA” pipeline in Spectronaut software (v18.3.230830.50606) [35] with default BGS Factory Settings mode. The protein database was downloaded from Uniprot database [36]. KNN algorithm was used to impute missing values. Quantitative data were median-normalized and log2-transformed for downstream analyses. The limma R package was used to detect differentially expressed proteins (DEPs) between groups. GO and KEGG enrichment analyses were performed using clusterProfiler R package (v4.8.3) [37] with a significance threshold set to 0.05. GOplot (v 1.0.2) [38], ggplot2 (v3.4.4) and enrichplot (v1.22.0) R packages were used to visualize GO and KEGG results. Pheatmap R package (v1.0.12) was used to generate heatmaps for DEPs based on their median-normalized expression levels.

### Statistical analysis

Data were analyzed with GraphPad 7.0 and presented as mean ± SD. The Student’s t-test was used for comparisons between the two groups. When more than two groups existed, statistical significance was assessed using one-way or two-way ANOVA, and Tukey test was conducted to determine the pairwise differences. The Kruskal-Wallis test was performed for data that were not normally distributed or if the variances of the groups were unequal. We considered *P* < 0.05 to be statistically significant.

## Results and discussion

### Synthesis and characterization of Au-CA NPs and Au-MEA NPs

**Fig. 1:**
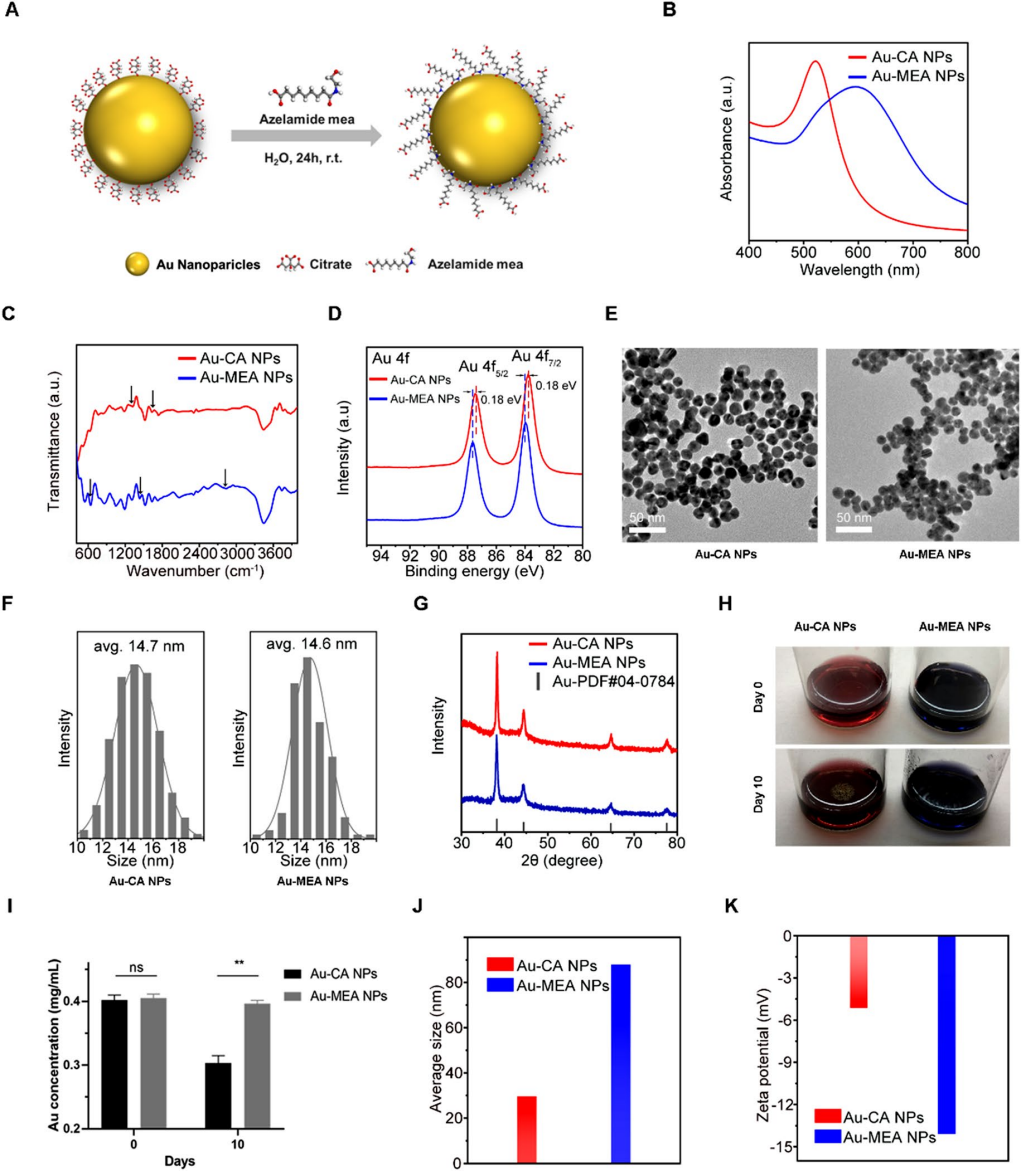
Synthesis and Characterization of Au-CA NPs and Au-MEA NPs. (**A**) Schematic illustration of Au-MEA NPs synthesis. (**B**) UV-vis absorption spectra of Au-CA NPs and Au-MEA NPs. (**C**) FTIR spectra of Au-CA NPs and Au-MEA NPs. (**D**) XPS spectra of Au-CA NPs and Au-MEA NPs. (**E**-**F**) TEM image of Au-CA NPs and Au-MEA NPs and their size distribution. Scale bar: 50 nm. (**G**) XRD of Au-CA NPs and Au-MEA NPs. (**H**) Representative images of Au-CA NPs and Au-MEA NPs aqueous solution at day 0 and gold aggregation in supernatant till 10 days. (**I**) Gold concentration in their supernatant tested by ICP-MS on day 0 or left at room temperature for 10 days. (**J**) DLS measurements for hydrodynamic diameter of Au-CA NPs and Au-MEA NPs. (**K**) Zeta potential of Au-CA NPs and Au-MEA NPs measured by DLS analysis. (**P < 0.01; ns, not significant; Student’s t-test was used.)

Firstly, the Au-CA NPs were prepared according to previously reported method with slight modification [32]. The Au-MEA NPs were produced via a facile ligand exchange process driven by concentration gradient (Fig. 1A). The UV-Vis spectrum of Au-CA NPs showed a characteristic Au NP absorption at about 521 nm, whereas the characteristic absorption peak position red-shifts to 594 nm after ligand substitution, which verified the successful attachment of azelamide MEA to the surface of Au NPs, (Fig. 1B). Furthermore, the Fourier transform infrared spectroscopy (FTIR) spectrum (Fig. 1C) of Au-CA NPs shows features originate from the surface-capped citrate ligands, such as the asymmetric C = O stretching vibration at 1627 cm^− 1^ and symmetric C-O stretching vibration at 1324 cm^− 1^. On the contrary, the Au-MEA NPs exhibit new spectrum features associated with C-H stretching vibration at 2932 cm^− 1^, C-N stretching vibration at 1428 cm^− 1^, and N-H wagging vibration at 644 cm^− 1^, which were bearing extensive similarity to that of the free azelamide MEA ligand (Figure S1). The surface chemical state of Au on two samples was elucidated by X-ray photoelectron spectroscopy (XPS) analysis. The Au 4f spectrum of Au-MEA NPs in Fig. 1D shows a positive shift (around 0.18 eV) in comparison with that of Au-CA NPs, indicating the electronic interaction between the Au NPs and the azelamide MEA ligand. These results together definitely demonstrate successful azelamide MEA ligand exchange.

The structure characterization by TEM (Fig. 1E-F) indicates that the Au-MEA NPs generated by the ligand exchange process possess morphology identical to that of Au-CA NP precursor and the size distribution of the two types of Au NPs is similar, with an average diameter of approximately 15 nm. The X-ray diffraction studies (Fig. 1G) shows that the particles all feature high crystallinity, which further demonstrates the absence of significant change in terms of both morphology and structure of particles during the ligand exchange.

Au-MEA NPs and Au-CA NPs aqueous solutions at room temperature for 10 days revealed that Au-MEA NPs in deionized water was more stable than Au-CA NPs as the aggregates of gold nanoparticles easily precipitated in Au-CA NPs solution (Fig. 1H). ICP-MS confirmed no appreciable reduction in the level of gold in the supernatant of the Au-MEA NPs aqueous solution at day 10 compared to day 0, whereas the gold remaining in the supernatant of the Au-CA NPs solution decreased appreciably after the suspension had been left for 10 days (Fig. 1I). The DLS analysis shows that the average size of Au-MEA NPs in aqueous solution is larger than that of Au-CA NPs. It may be attributed to the increase of strongly bound water shell generated by the continuous phase and the polymer on the Au-MEA NPs (Fig. 1J). Moreover, the zeta potential values of Au-CA NPs and Au-MEA NPs in DI water are − 5.15 mV and − 14.1 mV, respectively (Fig. 1K), suggesting that the Au-MEA NPs is more stable than Au-CA NPs in aqueous solution.

### Skin-penetrating ability of Au-MEA NPs gel

**Fig. 2:**
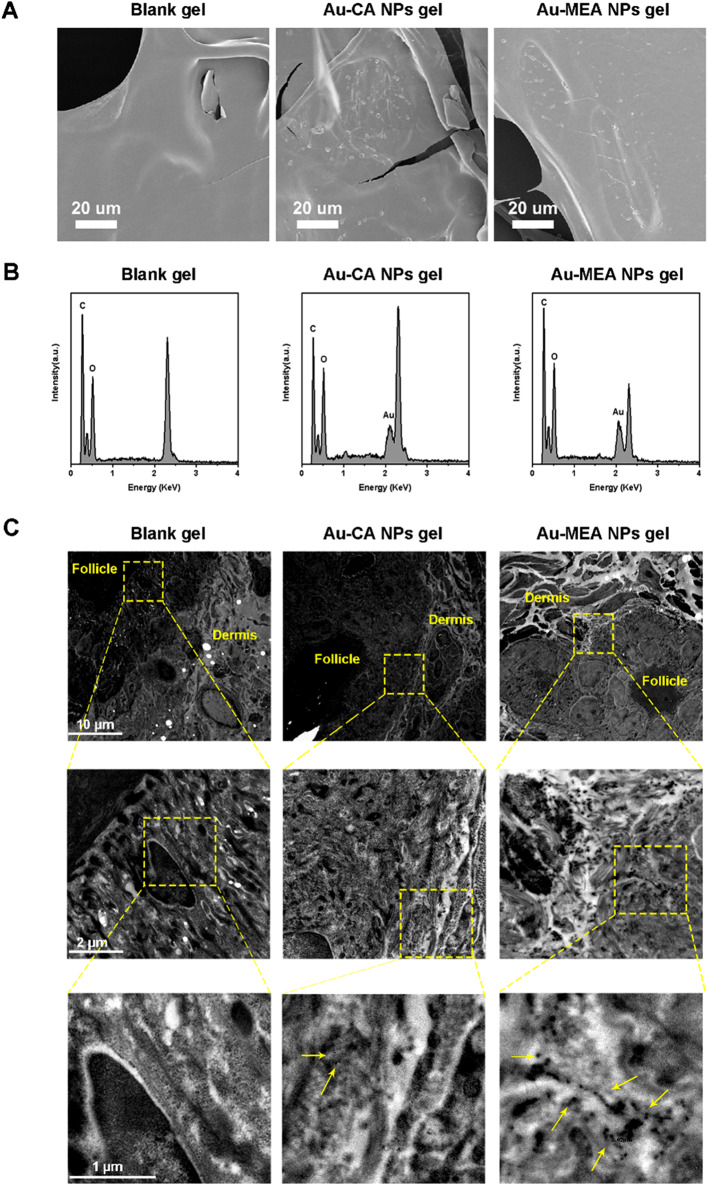
Skin penetration ability of AuNPs gel in mice. (**A**) SEM images of Blank gel and AuNPs gel. (**B**) The chemical composition of Blank gel and AuNPs gel. (**C**) TEM images of dorsal skin samples of mice treated with Blank gel, Au-CA NPs gel and Au-MEA NPs

Figure 2A shows the SEM images of the blank SEPIMAX ZEN gel (Blank gel), Au-CA NPs gel and Au-MEA NPs gel. Compared with the Blank gel, the surface of Au-CA NPs gel and Au-MEA NPs gel became rough and granular. The chemical composition of Blank gel and AuNPs gel was measured by energy-dispersive X-ray spectrometry (EDS). Elemental gold was detected only in Au-CA and Au-MEA NPs gel but not in Blank gel (Fig. 2B), further confirming the successful embedding of Au-CA and Au-MEA NPs in SEPIMAX ZEN gel.

Studies found that AuNPs can easily penetrate the stratum corneum (SC) and stratum lucidum of thick skin [39, 40]. Related studies demonstrate that AuNPs can permeate the human skin in a dose-dependent manner and reach the dermis, where these nanoparticles may be available for systemic diffusion [41, 42]. We next investigated the ability of Au-MEA NPs to penetrate the SC layer after transcutaneous application for 5 days. As expected, the control group showed no characteristic particles in the TEM skin section of mice (Fig. 2C). In Au-CA NPs group, a small amount of gold particles was found in the skin, possibly due to nanoparticle aggregation, resulting in minimal skin deposition. In the Au-MEA NPs group, the sample showed some accumulation of gold nanoparticles in the dermis. Several studies have confirmed that gold nanoparticles can penetrate into the stratum corneum and dermis of mice and rats, and even deeper layer of skin [43, 44]. In addition, the tiny size of gold nanoparticles owns the stronger skin permeability. Except the accumulation of gold nanoparticles in hair follicles, the intercellular pathway is considered to be the main pathway for nanoparticles to penetrate the skin. One study suggests that gold nanoparticles can create hydrophobic vacancies in the stratum corneum, which not only facilitate their own passage, but also facilitate the delivery of hydrophobic drugs [45]. Combined with the TEM results of the skin, we hypothesized that Au-MEA NPs penetrate into the skin via follicular or intercellular pathway. ICP-MS data showed that the Au-MEA NPs group had significantly higher levels in healthy skin than the Au-CA NPs group (Figure S3A).

### The treatment effects of Au-MEA NPs gel on imiquimod (IMQ) induced skin inflammation in mice

**Fig. 3:**
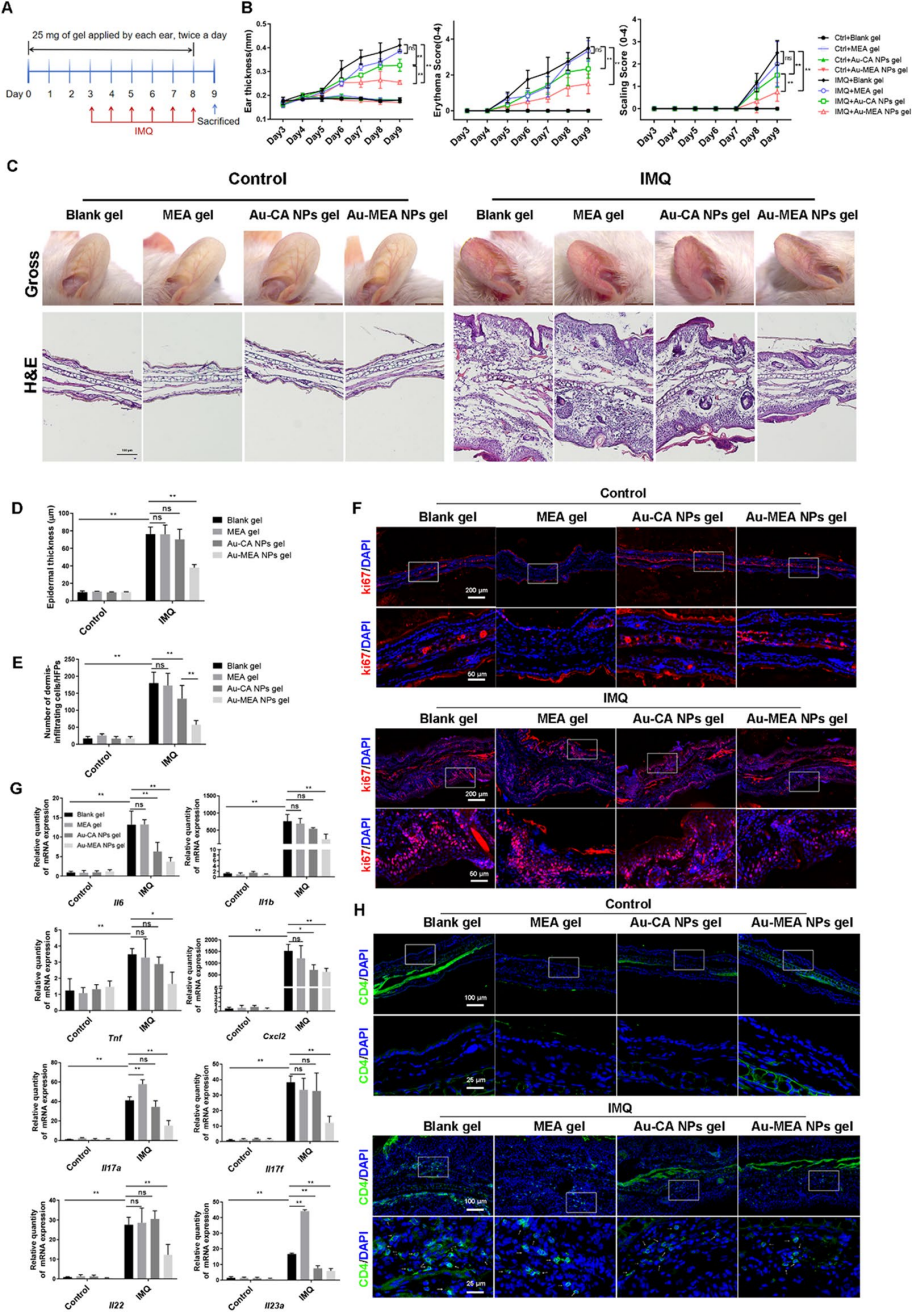
Topical application of Au-MEA NPs gel ameliorates psoriasiform skin inflammation. (**A**) Experimental protocol for transcutaneous application of Blank gel, MEA gel, Au-CA NPs gel, or Au-MEA NPs gel in the murine model of IMQ. (**B**) Clinical score of ear thickness, scaling, and erythema of psoriatic mice. (**C**) Representative phenotypic presentation and H&E staining of mouse ear treated with Blank gel, MEA gel, Au-CA NPs gel, or Au-MEA NPs gel. (**D**) Quantification of epidermal thickness. (**E**) The number of dermis-infiltrating cells in the ear tissues. (**F**) The Ki67 expression of mice ear treated with different gel in IMQ or control group were visualized by immunofluorescence. (**G**) The mRNA levels of inflammatory cytokines in tissues. (**H**) The CD4 positive T cells infiltration in mice ear treated with different gel in IMQ or control group. Data are presented as means ± SD (*P < 0.05, **P < 0.01; ns, not significant; 2-way ANOVA test was used.)

Based on the promising skin-penetrating ability of Au-MEA NPs, we investigated their efficacy after a transcutaneous administration against psoriasiform skin inflammation (Fig. 3). Compared with the model group, treatment with Au-MEA NPs attenuated the ear thickness, scaling and erythema in psoriatic mice, and showed better therapeutic efficacy than Au-CA NPs (Fig. 3B, C).

Histological analysis showed marked epidermal hyperplasia and the number of dermis-infiltrating cells, in IMQ-induced psoriasiform skin were improved in the Au-MEA NPs treated mice compared to Au-CA NPs treated mice (Fig. 3C-E). We found that keratinocyte proliferation was also reduced in Au-MEA NPs gel treated mice compared to Blank gel and Au-CA NPs gel treated mice (Fig. 3F and Figure S2A). In addition, the production of the psoriasis-related cytokines, *Il6*, *Il1b*, *Tnf*, *Cxcl2*, *Il17a*, *Il17f*, *Il22*, *Il23a*, and the infiltration of immune cells were significantly reduced by Au-MEA NPs, which were more effective than Au-CA NPs (Fig. 3G, H and Figure S2B). For in vivo experiments using IMQ-induced mice, transcutaneously administered MEA gel showed similar therapeutic efficacy to blank gel, indicating that the therapeutic efficacy of Au-MEA NPs gel on psoriasiform skin dermatitis is not due to the outermost azelamide MEA. Taken together, we demonstrated that these Au-MEA NPs effectively treated psoriasiform skin inflammation.

### The treatment effects of Au-MEA NPs gel on LL37-induced skin inflammation in mice

**Fig. 4:**
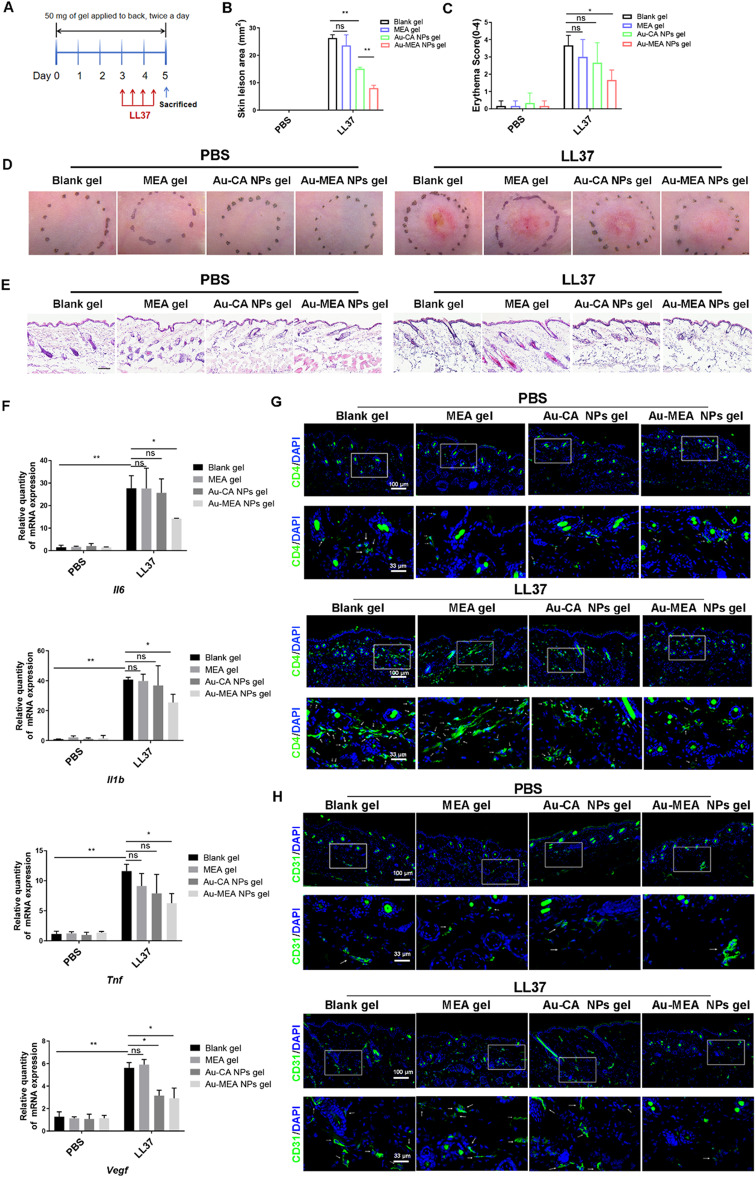
Au-MEA NPs gel alleviates rosacea-like development. (**A**) Experimental protocol for transcutaneous application of Blank gel, MEA gel, Au-CA NPs gel, or Au-MEA NPs gel in the murine model of rosacea. The effects of different gel on lesion area (**B**) and redness score (**C**) in rosacea-like mice. (**D**) Representative phenotypic manifestations of LL37 or PBS injected back skin of mice after percutaneous application of Blank gel, MEA gel, Au-CA NPs gel, or Au-MEA NPs gel. (**E**) H&E staining of mice skin lesion. Scale bars: 100 μm (**F**) The mRNA levels of *Tnf*, *Il6*, *Il1b* and *Vegf* in mice skin lesion. (**G**) Immunofluorescence of CD4^+^ T cells in mice skin treated with different gel in LL37 or PBS group. (**H**) Immunofluorescence of CD31^+^ microvascular in mice skin treated with different gel in LL37 or PBS group. Data are presented as means ± SD (*P < 0.05, **P < 0.01; ns, not significant; 2-way ANOVA test was used.)

Previous studies have shown that inflammatory cell infiltration and dermal vasculature dysfunction are significant features of rosacea [46, 47]. To dissect the therapeutic effect of Au-MEA NPs on rosacea, mice received transcutaneous application of Au-MEA NPs gel for 5 days (Fig. 4A). We detected that Au-MEA NPs gel significantly improved LL37-induced rosacea-like phenotypes compared with Blank gel and Au-CA NPs gel, including a lower redness score and area of erythema (Fig. 4B-D). H&E staining analysis revealed that inflammatory cell infiltration was significantly reduced in the rosacea-like mice treated with Au-MEA NPs, which were better than those treated with Au-CA NPs gel (Fig. 4E and Figure S2C). Compared with Blank gel group mice after LL37 injection, Au-MEA NPs suppressed the expression of proinflammatory cytokines, such as *Tnf*, *Il6*, *Il1b* and *Vegf* in rosacea-like dermatitis (Fig. 4F). Moreover, Au-MEA NPs treatment suggested higher suppressed efficacy against the expression of proinflammatory cytokines, compared with Au-CA NPs group (Fig. 4F). Consistent with these findings, immunofluorescence analysis showed that the number of CD31^+^ microvascular and CD4^+^ T-cell infiltration were significantly reduced in the Au-MEA NPs group compared with the Blank gel group and Au-CA NPs group after injection with LL37 (Fig. 4G, H and Figure S2D, E). Moreover, the transcutaneous application of MEA gel showed similar therapeutic effectiveness to Blank gel in LL37-induced mice, indicating that the therapeutic efficacy of Au-MEA NPs gel on rosacea-like skin dermatitis is not due to the outermost azelamide MEA. Collectively, these data demonstrate the momentous role of Au-MEA NPs in LL37-induced skin inflammation. More importantly, the therapeutic efficacy of Au-MEA NPs was found to be superior when compared with the Au-CA NPs.

### Proteomic analyses of the therapeutic mechanism of Au-MEA NPs gel in vivo

**Fig. 5:**
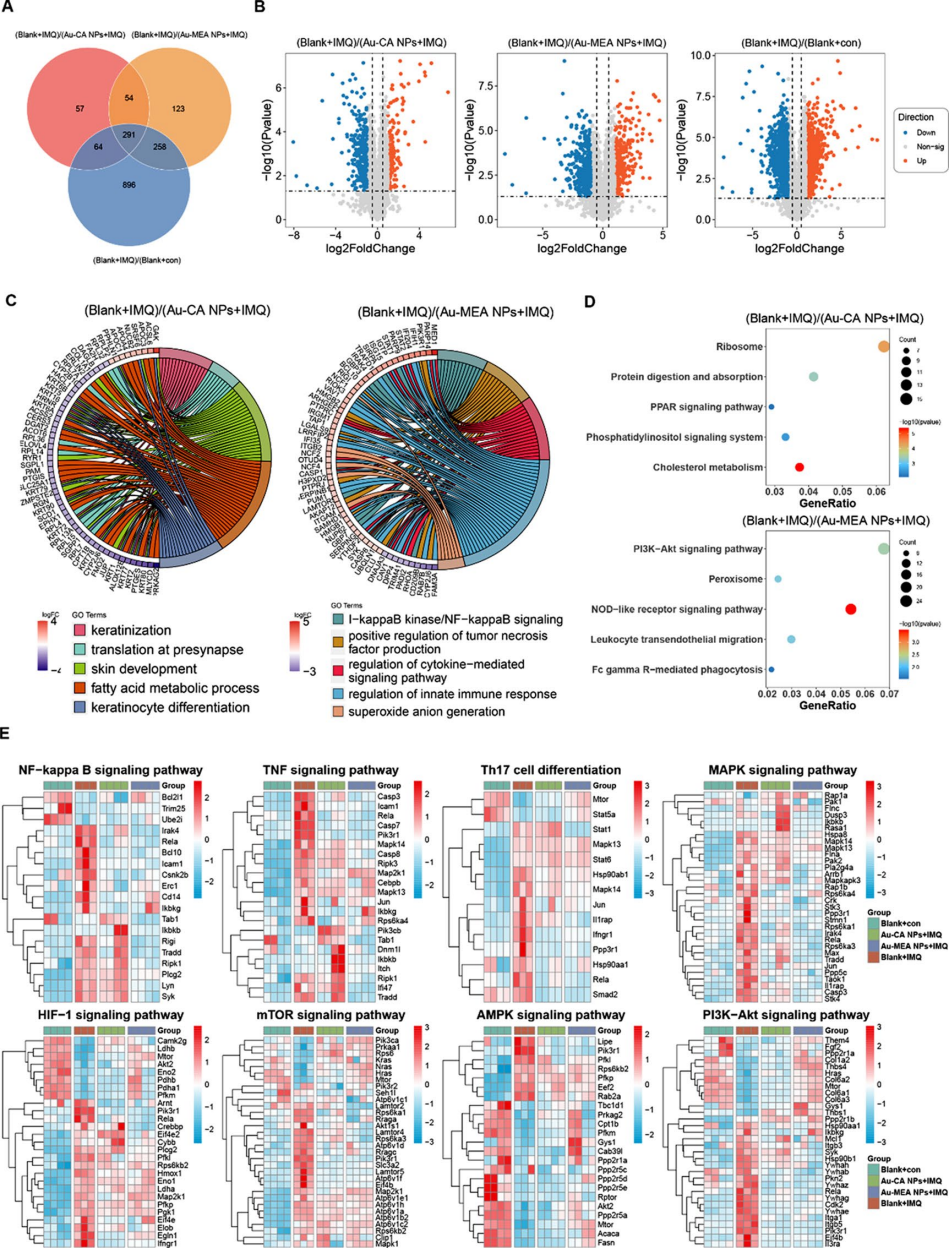
Proteomics analysis of IMQ-induced psoriasis in mice. (**A**) Venn diagram of proteomics analysis showing DEPs in pairs between the Blank + con, Blank + IMQ, Au-CA NPs + IMQ and Au-MEA NPs + IMQ group. (**B**) Volcano plots of DEPs identified between the Blank + IMQ and Au-CA NPs + IMQ group (left), the Blank + IMQ and Au-MEA NPs + IMQ group (middle) and the Blank + IMQ and Blank + con group (right). (**C**) Significantly enriched GO biological processes of the DEPs between the Blank + IMQ and Au-CA NPs + IMQ group (left) and the Blank + IMQ and Au-MEA NPs + IMQ group (right). (**D**) KEGG pathways of differential proteins between the Blank + IMQ and Au-CA NPs + IMQ group (top) and the Blank + IMQ and Au-MEA NPs + IMQ group (bottom). (**E**) Heat maps of DEPs in function categories including NF-kappa B signaling pathway, TNF signaling pathway, Th17 cell differentiation, MAPK signaling pathway, the HIF-1 signaling pathway, mTOR signaling pathway, AMPK signaling pathway, and PI3K-Akt signaling pathway (red indicating relatively high expressed proteins, blue indicating relatively low expressed proteins)

To further analyze the therapeutic effects of Au-MEA NPs on inflammatory skin diseases, we performed proteomic analyses on the above psoriasiform and rosacea-like skin lesion, respectively. In the IMQ-induced psoriasiform skin, we identified proteins with p-value < 0.05 & log2 (foldchange) ≥ 1 as DEPs. Venn diagrams showed significant differences in proteome profiles among the Blank + con group, Blank + IMQ group, Au-CA NPs + IMQ group and Au-MEA NPs + IMQ group (Fig. 5A). Compared with the Blank + con group, 1509 DEPs (709 up and 800 down) were observed in IMQ-induced psoriasiform skin (Fig. 5B). The 466 DEPs (189 up and 277 down) in Blank + IMQ group versus Au-CA NPs + IMQ group were enriched in skin development, fatty acid metabolism, process keratinocyte differentiation, and ribosome (Fig. 5B-D). The 726 DEPs (337 up and 389 down) in Blank + IMQ group versus Au-MEA NPs + IMQ group were enriched in inflammation-related pathways, including I-kappaB kinase/NF-kappaB signaling, positive regulation of tumor necrosis factor production, regulation of cytokine-mediated signaling pathway, PI3K-Akt signaling pathway, NOD-like receptor signaling pathway (Fig. 5B-D). Cluster analysis and heat mapping showed that Au-MEA NPs treatment reversed various inflammation-related signaling pathways, including NF-kappa B signaling pathway, TNF signaling pathway, Th17 cell differentiation, MAPK signaling pathway, the HIF-1 signaling pathway, mTOR signaling pathway, AMPK signaling pathway, and PI3K-Akt signaling pathway to near normal levels (Fig. 5E).

**Fig. 6:**
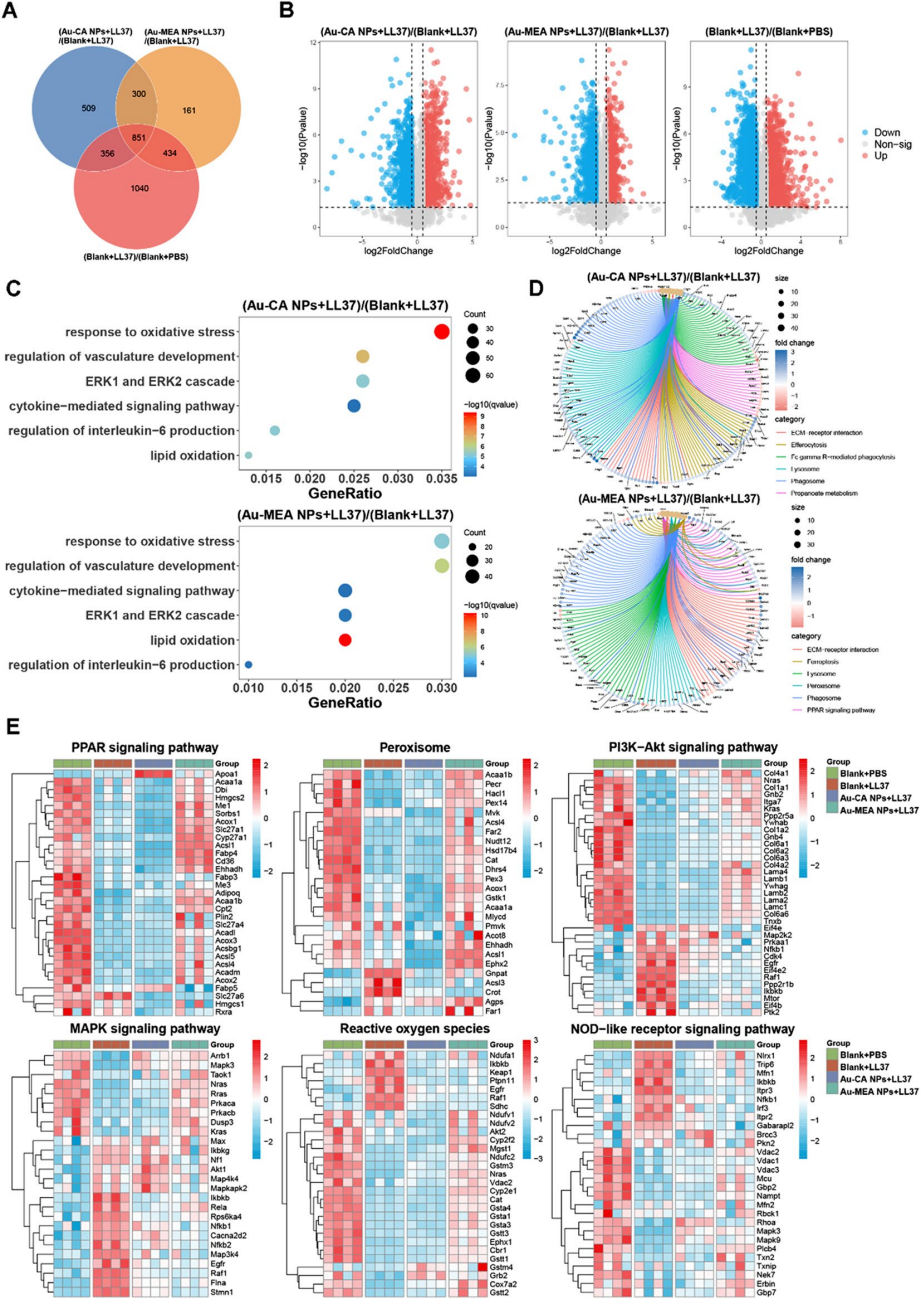
Proteomics analysis of LL37-induced rosacea in mice. (**A**) Venn diagram of proteomics analysis showing DEPs in pairs between the Blank + PBS, Blank + LL37, Au-CA NPs + LL37 and Au-MEA NPs + LL37 group. (**B**) Volcano plots of DEPs identified between the Au-CA NPs + LL37 and Blank + LL37 group (left), the Au-MEA NPs + LL37 and Blank + LL37 group (middle), the Blank + LL37 and Blank + PBS group (right). (**C**) Significantly enriched GO biological processes of the DEPs between the Au-CA NPs + LL37 and Blank + LL37 group (top) and the Au-MEA NPs + LL37 and Blank + LL37 group (bottom). (**D**) KEGG pathways of differential proteins between the Au-CA NPs + LL37 and Blank + LL37 group (top) and the Au-MEA NPs + LL37 and Blank + LL37 group (bottom). (**E**) Heat maps of DEPs in function categories including PI3K-Akt signaling pathway, MAPK signaling pathway, reactive oxygen species, peroxisome, PPAR signaling pathway, and NOD-like receptor signaling pathway (red indicating relatively high expressed proteins, blue indicating relatively low expressed proteins)

In the LL37-induced mouse rosacea model, differential expression analysis was performed and proteins with p-value < 0.05 & log2(foldchange) ≥ 0.5 were identified as DEPs. Venn diagrams revealed significant differences in proteomic profiles between the Blank + PBS group, Blank + LL37 group, Au-CA NPs + LL37 group, and Au-MEA NPs + LL37 group (Fig. 6A). 2681 DEPs (1282 up and 1399 down) were differentially expressed in rosacea-like skin compared to control skin (Fig. 6B). For Au-CA NPs + LL37 group versus Blank + LL37 group comparisons, 2016 DEPs were identified (1035 up and 981 down) (Fig. 6B). A total of 1746 DEPs (895 up and 851 genes down) were identified when comparing Au-MEA NPs + LL37 group with Blank + LL37 group (Fig. 6B). Pathways that were significantly enriched in the Au-CA NPs + LL37 group relative to the Blank + LL37 group and Au-MEA NPs + LL37 relative to the Blank + LL37 were shown in Fig. 6C-D. GO enrichment analysis showed that both Au-CA NPs and Au-MEA NPs treatments were involved in oxidative stress, lipid oxidation, regulation of vasculature development, cytokine-mediated signaling pathway, regulation of interleukin-6 production when comparing with Blank + LL37 group (Fig. 6C). KEGG analysis showed that both Au-CA NPs and Au-MEA NPs treatments had effects on biological processes such as ECM-receptor interaction, lysosome, and phagosome when compared with the Blank + LL37 group (Fig. 6D). In addition, Au-MEA NPs treatment also regulated the peroxisome and PPAR signaling pathway, compared with the Blank + LL37 group (Fig. 6D). Cluster analysis and heatmap showed that Au-MEA NPs treatment reversed multiple inflammatory and oxidative stress-related signaling pathways, including PI3K-Akt signaling pathway, MAPK signaling pathway, reactive oxygen species, peroxisome, PPAR signaling pathway, and NOD-like receptor signaling pathway to near normal levels (Fig. 6E).

Thus, proteomic analysis further revealed that Au-MEA NPs may treat inflammatory skin diseases by affecting the activation of inflammatory and oxidative stress-related pathways.

### In vivo safety and tissue distribution of Au-MEA NPs

To verify the safety of Au NPs in mice, the mice in different treatment groups were dissected after modeling, and the heart, liver, spleen, lung and kidney were collected for H&E staining and ICP-MS, respectively. Compared with the control group, the histological state of the major organs of the mice in the Au-CA or Au-MEA NPs gel treatment group did not indicate any abnormal changes (Figure S3B). ICP-MS analysis revealed that gold was mainly accumulated in the skin tissues after Au-CA NPs or Au-MEA NPs gel treatment. There were no significant differences in the organs in Au-CA NPs or Au-MEA NPs group including lung, liver, spleen, kidney, and heart compared with the control group (Figure S3A). In addition, hemolysis tests showed that the presence of large amounts of hemoglobin in the supernatant was observed only in the positive control tube (Figure S3C). All of the above findings suggest that these AuNPs gel have a good safety for skin therapy.

### Anti-inflammatory of Au-MEA NPs in vitro

As keratinocyte-mediated secretion of inflammatory cytokines and chemokines plays a momentous role in the progression of rosacea and psoriasis [33, 48–50]. Therefore, we explored the actions of Au NPs on these pro-inflammatory factors in TNF-α-treated HaCaTcells model. First, we performed CCK-8 analysis to investigate the effect of Au NPs on HaCaT viability. We found that different concentrations of Au-CA NPs or Au-MEA NPs (5 µg/mL-100 µg/mL) had no significant effects on the viability of HaCaT cells (Figure S4A) and we chose a concentration of 25 µg/mL in cell experiment. Huo et al. found that 16 nm Au NPs were located in the cytoplasm of MCF-7 cells after 24 h treatment [51]. Given the cell-penetrating properties of this size dimension, we sought to determine whether Au-MEA NPs could also be internalized by skin cells. To this end, we incubated HaCaT cells with FITC-labeled Au NPs for various times and analyzed intracellular fluorescence using fluorescence microscope. As expected, the intracellular fluorescence intensity gradually increased with increasing incubation time, reaching a maximum at 24 h (results observed). FITC-labeled Au NPs were distributed in the cytoplasmic compartments of HaCaT cells (Figure S4B). ICP-MS showed that the amount of gold in HaCaT cells treated with Au-MEA NPs or Au-CA NPs was much higher than that in solvent-treated control cells, indicating the successful internalization of Au-MEA NPs and Au-CA NPs. And there were similar gold contents between Au-MEA NPs and Au-CA NPs (Figure S4C). To study the distribution of AuNPs in cells, HaCaT cells were processed for TEM after exposure to Au-MEA NPs or Au-CA NPs for 24 h. Solvent-treated cells showed no abnormalities (Figure S4D), whereas Au-MEA NPs- or Au-CA NPs-treated HaCaT cells showed endosomes with a large number of small particles inside and such endosomes were mainly observed near the cell membrane, suggesting that AuNPs may enter the cells via membrane endocytosis. A number of particles were also deposited in lysosomes, mitochondria and near the nuclear membrane of the treated cells (Figure S4D). Taken together, these results showed that the approximately 15 nm AuNPs were able to enter the cytoplasm and did not have a significant cytotoxic effect.

**Fig. 7:**
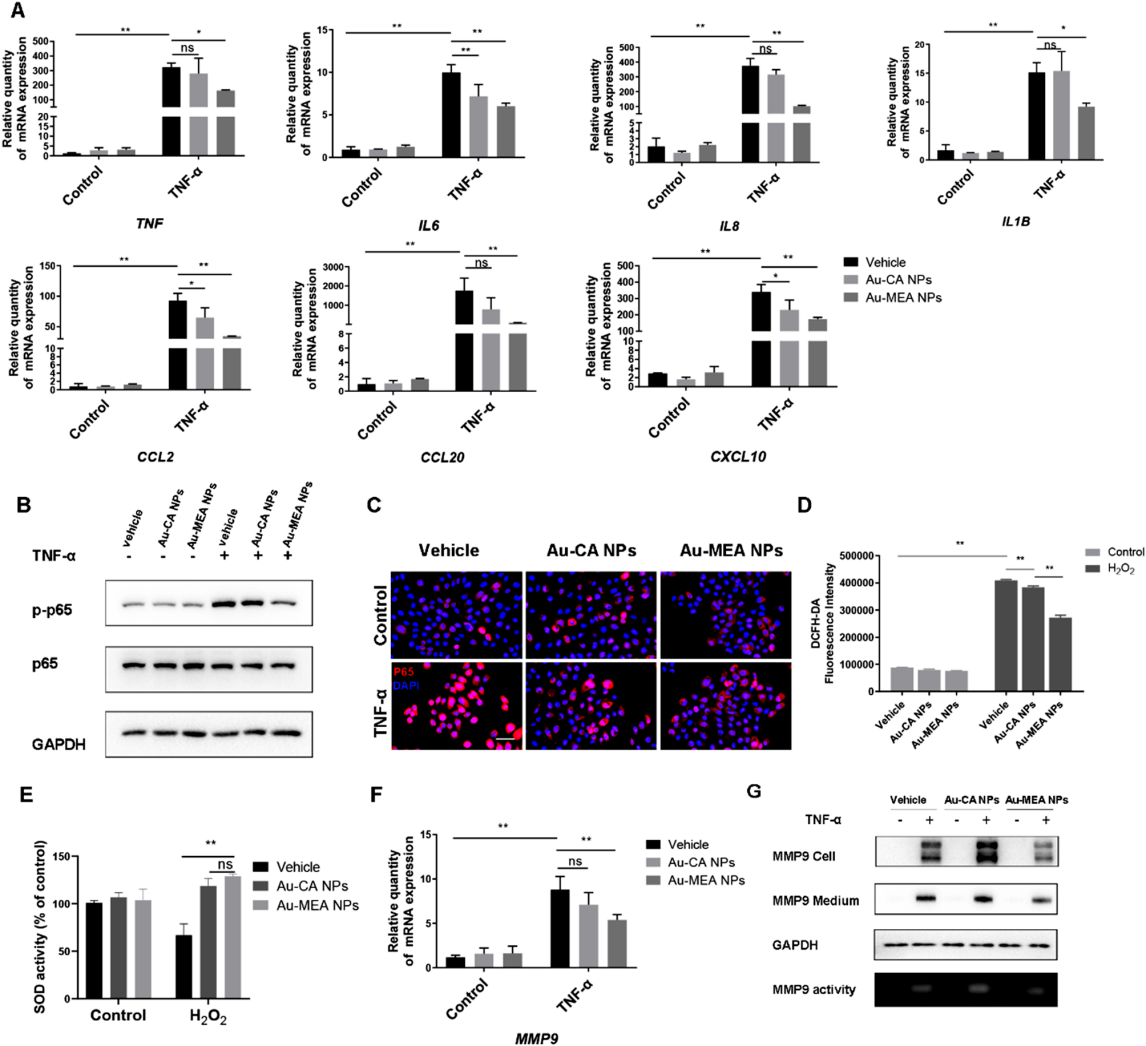
Au-MEA NPs attenuates cytokine production in vitro. (**A**) The mRNA expression levels of *TNF*, *IL6*, *IL8*, *IL1B*, *CCL2*, *CCL20* and *CXCL10* in HaCaT cells. (**B**) Western blotting of p-p65 and p65 in cell lysates from vehicle and AuNPs-pretreated HaCaT cells. (**C**) Immunofluorescence of p65 in AuNPs-pretreated HaCaT cells. Scale bars: 50 μm. (**D**) The ROS levels of HaCaT cells treated with H_2_O_2_. (**E**) The activity of SOD in HaCaT cells treated with H_2_O_2_. (**F**) The mRNA levels of *MMP9* by quantitative real-time RT-PCR. (**G**) Western-blot for MMP9 and gelatin zymography showing MMP9 activity. Data are presented as means ± SD (*P < 0.05, **P < 0.01; ns, not significant; 2-way ANOVA test was used)

The overexpression of pro-inflammatory cytokines secreted by keratinocytes contributes to the pathogenesis of rosacea and psoriasis [52, 53]. In this study, Au-MEA NPs suppressed TNF-α-induced upregulation of inflammatory factors including *TNF*, *IL6*, *IL8*, *IL1B*, *CCL2*, *CCL20* and *CXCL10* in HaCaT cells (Fig. 7A). NF-κB signaling has been implicated in a variety of inflammatory skin diseases, including psoriasis and rosacea, and is required for the production of several cytokines and chemokines [54–56]. Next, we detected the downstream NF-κB p65 in HaCaT cells. Here, we found that Au-MEA NPs repressed the activation of NF-κB signaling in TNF-α-induced inflammatory keratinocytes (Fig. 7B). Moreover, Au-MEA NPs treatment significantly inhibited TNF-α-induced NF-κB p65 nuclear translocation in HaCaT cells as determined by immunofluorescence (Fig. 7C).

Oxidative stress plays a significant role in the inflammatory skin diseases, and suppression of the ROS production rescued skin inflammation [9]. Metal nanomaterials have been reported to act as modulators of enzyme activity to exert antioxidant effects and participate in regulating inflammation [57]. To further investigate the anti-inflammatory mechanism of Au-MEA NPs in inflammatory skin diseases, we investigated the effect of Au-MEA NPs on key enzyme activities in inflammatory skin diseases. As shown in Fig. 7D, the H_2_O_2_-induced ROS accumulation was markedly reduced in AuNPs-treated groups, and Au-MEA NPs had a better inhibitory effect than Au-CA NPs. Besides, Au-MEA NPs could scavenge ROS through high-performance SOD activity (Fig. 7E). As a metal matrix protease, MMP9 plays an important role in various physiological processes such as inflammation and angiogenesis [19, 58, 59]. Therefore, we then investigated the change of the MMP9. As shown in Fig. 7F, the mRNA level of *MMP9* in TNF-α-stimulated HaCaT cells was inhibited by Au-MEA NPs, indicating that Au-MEA NPs could inhibit the transcription of MMP9. The expression level of MMP9 protein was further determined. Western-blot results showed that Au-MEA NPs could significantly suppress the expression level of MMP9 protein in cells and secreted by cells (Fig. 7G). To investigate the activation of MMP9 in HaCaT cells, we collected the medium and performed the gel zymography after TNF-α exposure (Fig. 7G). The active form of MMP9 was inhibited by Au-MEA NPs whereas a significant upregulation was induced by Au-CA NPs.

In a word, these data demonstrated the ability of Au-MEA NPs to eliminate keratinocyte-mediated inflammatory cytokines secretion, mainly by regulating the activity of SOD and MMP9.

## Conclusion

In summary, we synthesized Au-MEA NPs by ligand exchange method for the first time to effectively reduce psoriasiform and rosacea-like chronic skin inflammation. Although its long-term toxicity, penetration mechanism and penetration depth remain unclear. There is still a lot of work to be done in the future, such as further investigation and optimization of therapeutic dose and treatment frequency. In addition, further comparative studies with existing commercial gels are necessary for the evaluation and improvement of its performance. Notably, Au-MEA NPs has great clinical translation value in the treatment of inflammatory skin diseases because it represents a simple, safe, and effective therapy, and provides a unique inspiration and reference for the treatment of other diseases with similar pathological characteristics.

## Electronic supplementary material

Below is the link to the electronic supplementary material.

Supplementary Material 1

## Data Availability

No datasets were generated or analysed during the current study.

## Abbreviations

DAPI: 4′ 6-Diamidino-2-phenylindole
DEPs: Differentially expressed proteins
DLS: Dynamic light scattering
EDC: Ethylcarbodiimide hydrochloride
FBS: Fetal bovine serum
FTIR: Fourier transform infrared spectroscopy
IMQ: Imiquimod
MMPs: Matrix metalloproteinases
MPA: Mercaptopropionic acid
MEA: Monoethanolamine
NPs: Nanoparticles
NHS: N-hydroxysuccinimide
NF-κB: Nuclear factor kappa B
PFA: Paraformaldehyde
PVDF: Polyvinylidene fluoride
ROS: Reactive oxygen species
SEM: Scanning electron microscope
SC: Stratum corneum
SOD: Superoxide dismutase
TEM: Transmission electron microscopy
TNF-α: Tumor necrosis factor alpha
XPS: X-ray photoelectron spectroscopy
