# Supplementary material for: Skin-permeable gold nanoparticles with modifications azelamide monoethanolamine ameliorate inflammatory skin diseases
Source: Biomark Res. 2024 Oct 9;12:118. doi: 10.1186/s40364-024-00663-0 (PMC11465885; doi:10.1186/s40364-024-00663-0)
Supplement: Supplementary file 1 — Supplementary Material 1 [file 40364_2024_663_MOESM1_ESM.docx]

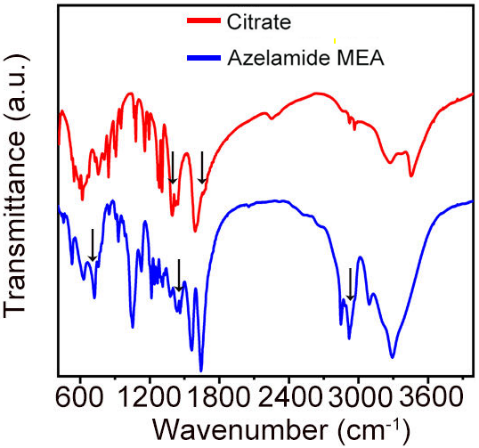


Figure S1. FTIR of citrate and azelamide MEA. The arrows highlighted the emerged peak of azelamide MEA (including C-H stretching vibration at 2932 cm^-1^, C-N stretching vibration at 1428 cm^-1^, and N-H wagging vibration at 644 cm^-1^), which were different from citrate.


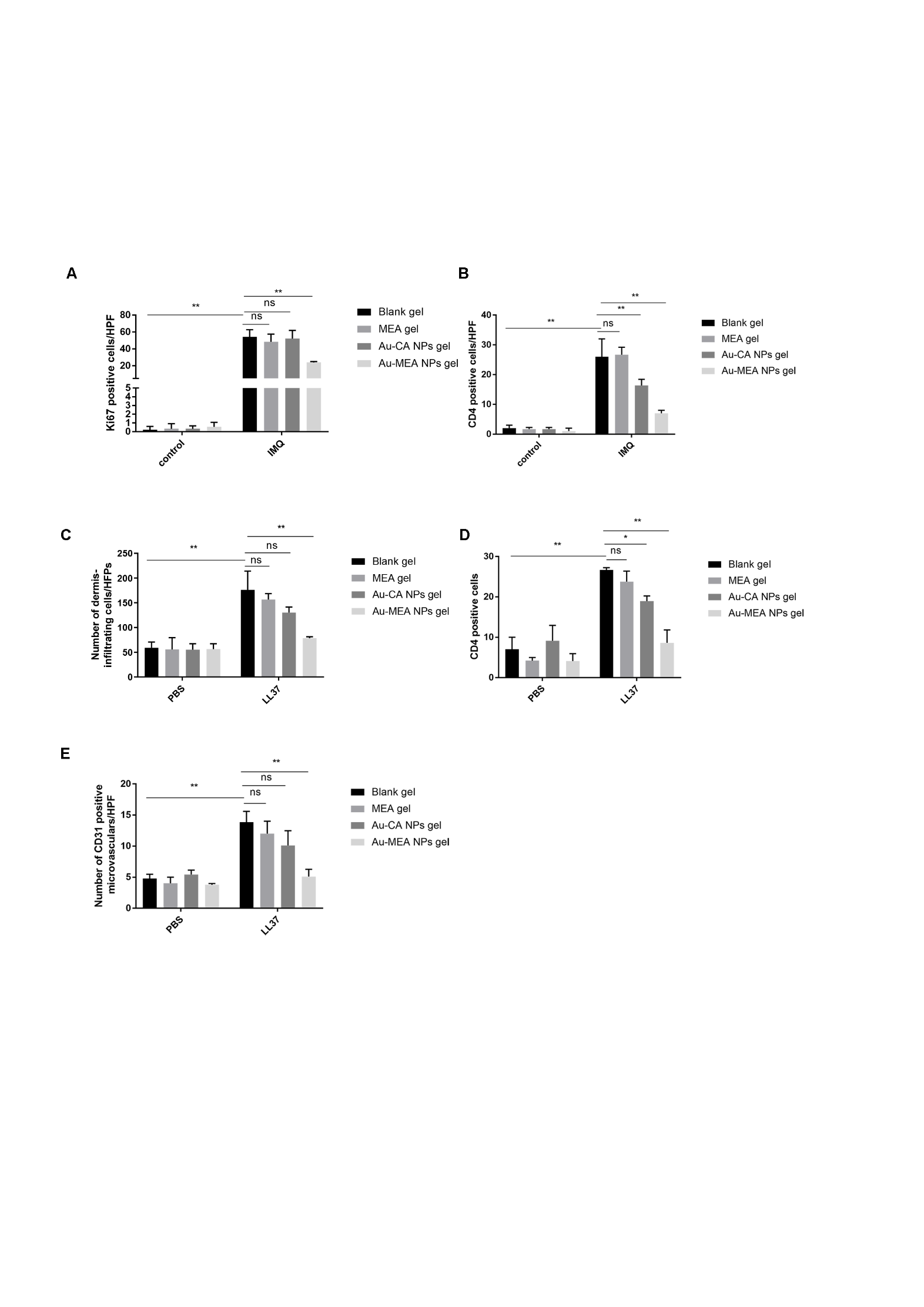


Figure S2. The number of positive cells in the skin tissues stained by Immunofluorescence. (A) The quantities of Ki67-positive cells in mice treated with IMQ/Control. (B) The quantities of CD4-positive cells in mice treated with IMQ/Control. (C) The number of dermis-infiltrating cells in the skin tissues. (D) The quantities of CD4 positive cells in mice treated with LL37/PBS. (E) The quantities of CD31-positive microvascular in mice treated with LL37/PBS. Data are presented as means ± SD (*P < 0.05, **P < 0.01; ns, not significant; 2-way ANOVA test was used.).


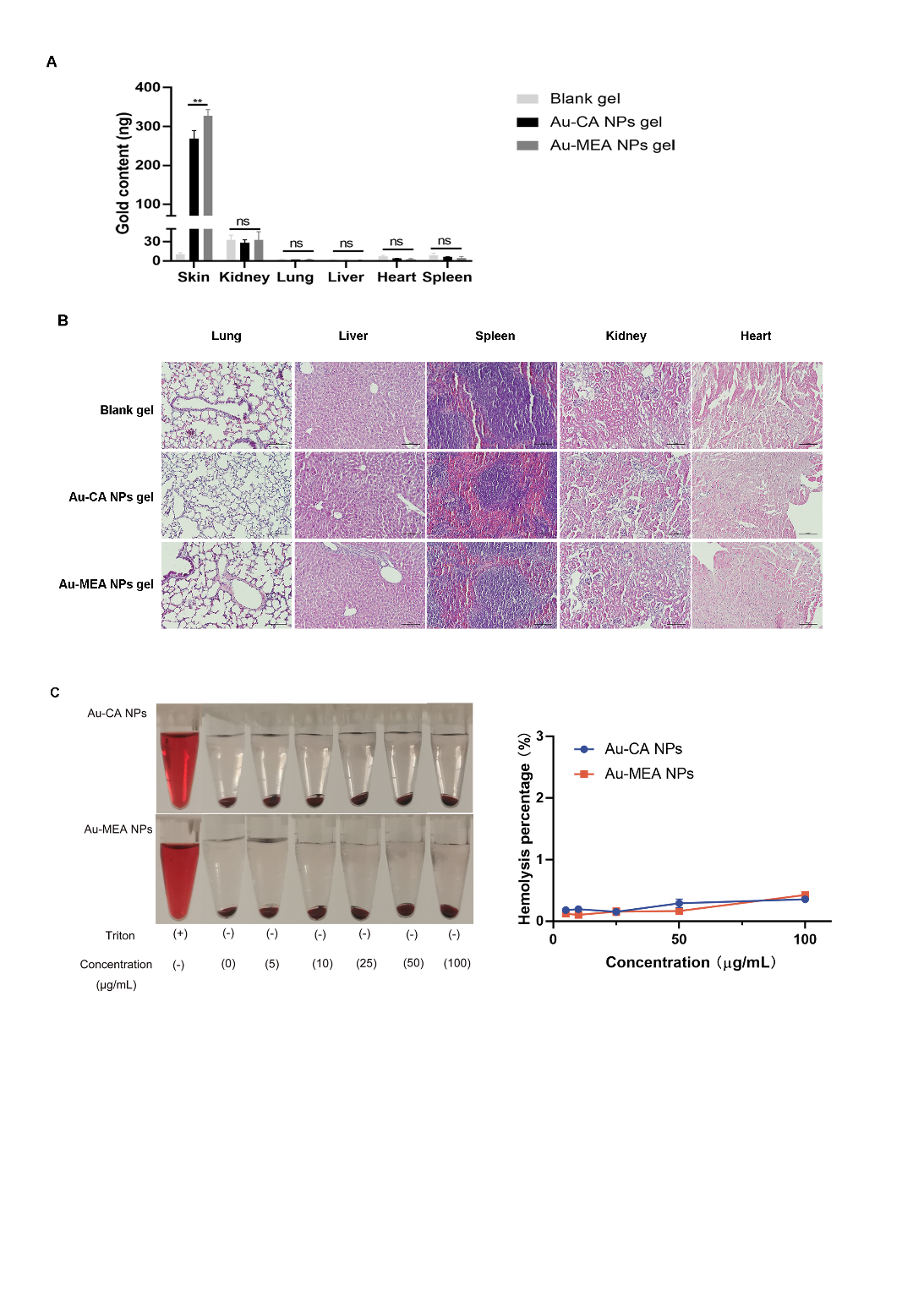


Figure S3. Biocompatibility, safety, and tissue distribution of AuNPs gel. (A) ICP-MS analysis of gold contents in skin, heart, liver, spleen, kidney, and lung from mice received transcutaneous application. (B) H&E staining images of heart, liver, spleen, kidney, and lung sections from mice received transcutaneous application. (C) Relative rate of hemolysis following 2 h incubation with different concentrations of Au-CA NPs and Au-MEA NPs suspension at 37 ℃. Image of samples after centrifugation at 13000 rpm for 10 min: 0 μg/mL (negative control), Triton (positive control), and nanoparticle suspension (5, 10, 25 ,50 and 100 μg/mL). Data are presented as means ± SD (*P < 0.05, **P < 0.01; ns, not significant; one-way ANOVA test was used.).


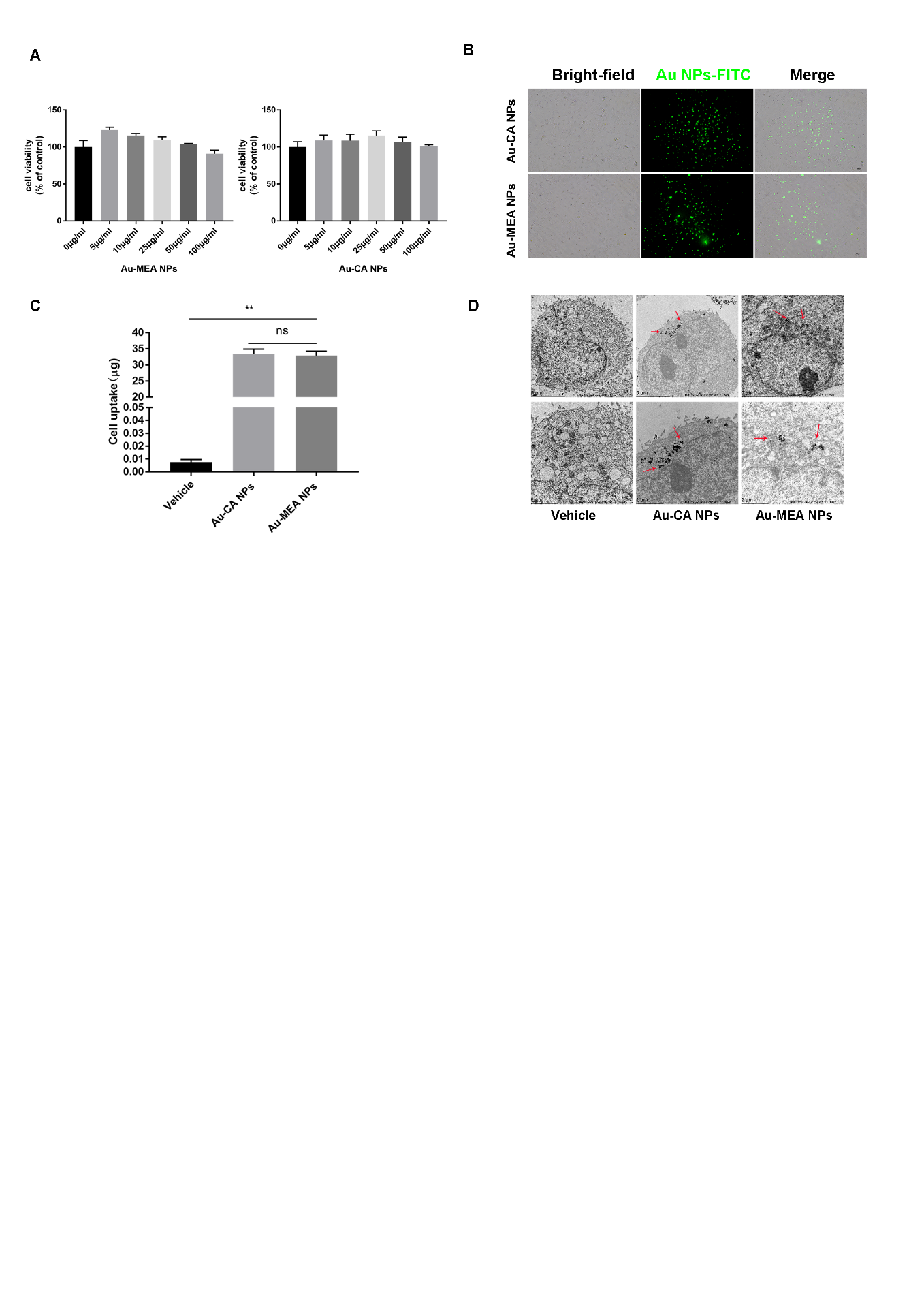
Figure S4. The cellular uptake of AuNPs in keratinocytes. (A) Viability of HaCaT cells following incubation with Au-CA NPs and Au-MEA NPs at the indicated concentrations for 24 h. The viability of AuNPs-treated cells was expressed as a percentage of control cell viability. (B) Intracellular uptake and localization of FITC-labeled Au-CA NPs and Au-MEA NPs in HaCaT cells. (C) ICP-MS analysis of gold contents in HaCaT cells. (D) TEM images of HaCaT cells treated with Au-CA NPs, Au-MEA NPs or vehicle for 24 h. Data are presented as means ± SD (*P < 0.05, **P < 0.01; ns, not significant; one-way ANOVA test or Kruskal-Wallis test was used.).
